# Location, Location, Location: The Role of Nuclear Positioning in the Repair of Collapsed Forks and Protection of Genome Stability

**DOI:** 10.3390/genes11060635

**Published:** 2020-06-09

**Authors:** Jenna M. Whalen, Catherine H. Freudenreich

**Affiliations:** 1 Department of Biology, Tufts University, Medford, MA 02155, USA; jenna.whalen@tufts.edu; 2 Program in Genetics, Tufts University, Boston, MA 02111, USA

**Keywords:** replication fork, fork collapse, replication fork barriers, fork restart, nuclear pore complex, sumoylation

## Abstract

Components of the nuclear pore complex (NPC) have been shown to play a crucial role in protecting against replication stress, and recovery from some types of stalled or collapsed replication forks requires movement of the DNA to the NPC in order to maintain genome stability. The role that nuclear positioning has on DNA repair has been investigated in several systems that inhibit normal replication. These include structure forming sequences (expanded CAG repeats), protein mediated stalls (replication fork barriers (RFBs)), stalls within the telomere sequence, and the use of drugs known to stall or collapse replication forks (HU + MMS or aphidicolin). Recently, the mechanism of relocation for collapsed replication forks to the NPC has been elucidated. Here, we will review the types of replication stress that relocate to the NPC, the current models for the mechanism of relocation, and the currently known protective effects of this movement.

## 1. Introduction

DNA must be accurately and efficiently replicated once during each cell cycle in order to maintain the genome. The replication fork normally travels about 1–2 kb/min in eukaryotic cells [1]. However, when a barrier to replication is encountered, this can lead to pausing of the replication fork, termed fork stalling. These barriers are usually called replication fork barriers (RFBs). The replication fork encounters many types of replication fork barriers during normal replication. Some examples of these are DNA secondary structures, DNA binding proteins, collisions with the transcription machinery, and damaged DNA bases [2,3]. An important characteristic of a stalled fork is that it still maintains the ability to resume replication once the barrier has been overcome [4]. The replisome may remain bound at the replication fork either in full or in part, depending on the damage situation. Even if replication cannot be restarted, the nascent DNA can be protected by the replisome remaining bound to the DNA until other restart pathways can be initiated. Examples of these alternative restart pathways are convergence with an oncoming fork from an active origin (or the firing of a dormant origin) [5,6,7], re-priming downstream of damaged DNA bases followed by filling in of the ssDNA gaps left behind [8,9], and a homologous recombination-dependent fork restart also known as recombination-dependent replication (RDR) [10].

Unfortunately, a stalled replication fork cannot always be simply restarted or rescued with an oncoming fork, especially in areas of the genome that have less replication origins or weak origins. If not restarted or rescued, or if the intra-S checkpoint is not activated, the stalled replication fork can turn into something more severe, a collapsed replication fork. The simplest definition of a collapsed fork is a replication fork that no longer has the ability to replicate DNA [11]. The field has further defined fork collapse two main ways: (1) the dissociation of the replisome and associated factors from the replication fork and (2) breakage or cleavage of the stalled replication fork. There is evidence for both definitions; however, there is no consensus. For example, there is evidence that the replisome components (e.g., Polε, Polα, and MCM helicase components) dissociate from the replication fork in the absence of proper DNA damage checkpoint activation [12,13,14,15]. However, another study showed that the replisome was still present at the stalled replication fork in checkpoint mutants but that the checkpoint is needed to keep the replisome functional via phosphorylation of the replicative helicase [16]. In the case of double-strand breaks (DSBs) at the replication fork, these could form by cleavage of structures formed at the stalled replication fork by endonucleases. An example of a structure that could form is a reversed fork. These are commonly formed in response to replication stress, especially in areas of the genome that are difficult to replicate or have DNA damage [11]. Reversed forks can be cleaved by endonucleases, such as Mus81 or the Slx4 complex, resulting in a DSB at the replication fork [11]. There is still some uncertainty as to how to define a collapsed fork (in fact, both definitions could be occurring at the same time), but in both cases described, the replication fork would no longer have the ability to replicate DNA, which has severe consequences. If the collapsed fork does not remain stable until an oncoming fork arrives, or if it is in an area with widely spaced or no origins (for example at a telomere), the fork collapse could result in unfinished DNA replication, which is very deleterious for the genome. Alternatively, cells could attempt to repair or restart the collapsed fork through homologous recombination (HR)-mediated mechanisms such as RDR. Although this can also result in increased genome instability, it is generally better than leaving DNA unreplicated [10,17]. It is therefore very important to understand what contributes to promoting fork restart and repair of collapsed replication forks.

## 2. Replication Stress and the Nuclear Pore Complex (NPC)

The nuclear pore complex (NPC) has been shown to be an area very important for the maintenance of genome stability after fork collapse, which is a function outside its normal role in the selective transport of proteins and RNA between the nucleus and the cytoplasm [18]. The largest subcomplex of the nuclear pore, the Y-shaped Nup84 complex, [19] in particular, plays a role in dealing with replication stress. This complex is made up of Nup84, Nup85, Nup120, Nup133, Nup145C, Sec13, and Seh1 in *Saccharomyces cerevisiae* [19]. In *Schizosaccharomyces pombe*, the complex is made up of Nup107, Nup85, Nup120, Nup133a, Nup133b, and Seh1 [20], and in humans, it is made up of Nup107, Nup85, Nup160, Nup133, Nup96, and Seh1 [19]. When components of this complex are deleted (e.g., *nup84Δ* in *S. cerevisiae*) cells are hypersensitive to DNA-damaging agents that can interfere with replication (e.g., HU, MMS, CPT, Phleomycin, and 4-NQO) [21,22,23]. Additionally, *nup84Δ* cells in *S. cerevisiae* are sensitive to caffeine [23], which inhibits the DNA damage checkpoint, which is relevant, since the checkpoint helps prevent fork collapse after fork stalling. In human cells, the mutation of Nup153 (Nup1 in *S. cerevisiae*) causes ionizing radiation (IR) sensitivity, and the mutation of Nup188 (Nup188 in *S. cerevisiae*) causes sensitivity to ATR inhibition [24]. Loss of another member of the Y-complex, Nup133, is synthetically lethal with genes involved in replication (e.g., Rad27) [25]. The authors proposed that the synthetic lethality could be due to a synergistic occurrence of DNA damage from excessive replication impairment by lack of both Nup133 and Rad27 [25]. In human cells, the knockdown of Nup133, Nup107, or Seh1 leads to an accumulation of spontaneous DNA damage [26]. Additionally, in mammalian cells, replication forks were shown to interact with nuclear pore complex components when under replication stress (personal communication, Dr. Anja Bielinsky). Overall, it seems that, in cases where cells are dealing with replication stress, it is important to also have components of the nuclear pore Y-complex, pointing to the complex playing a role in dealing with the replication stress.

## 3. Links between Replication Stress and the NPC: Sumoylation

The NPC is also a center for sumoylation regulation. Sumoylation is the covalent addition of SUMO (small ubiquitin-like modifier) to lysine residues of proteins. Sumoylation of target proteins is mediated by Ubc9, the SUMO E2 conjugating enzyme, and three SUMO E3 ligases in budding yeast: Mms21/Nse2, Siz1, and Siz2. These are homologous to Nse2 and Pli1 in *S. pombe*, Nse2 and PIAS in humans, and Cervantes/Quijote and dPIAS in *Drosophila* [27,28,29]. In yeast, the SUMO-protease Ulp1, which can remove SUMO from proteins, is anchored to the nuclear basket protein Nup60 [30]. Additionally, SUMO targeted ubiquitin ligase complexes (STUbLs) such as Slx5/Slx8 in *S. cerevisiae*, Rfp1/2 and Slx8 in *S. pombe*, Dgrn in *Drosophila*, and RNF4 and RNF11 in humans localize to the NPC via interaction with Nup84 (or its homologs) [31,32,33,34,35]. In addition to several proteins involved in sumoylation regulation localizing to the NPC, when these genes and others in the SUMO pathway are deleted, yeast cells are sensitive to DNA-damaging agents (e.g., Mms21, Siz1/2, Ubc9, Slx5/8, and Ulp1) [36]. The deletion of Slx5/8 in *S. pombe* led to hypersensitivity to DNA-damaging agents HU and MMS [31], and the mutation of Ulp1 also led to the accumulation of ssDNA during the S-phase [37]. If Ulp1 is prevented from localizing to the nuclear pore, there is an increase in genome instability, indicating that its function is tied to its NPC location [30]. Additionally, the genome instability seen in NPC mutants (Nup84 and Mlp1/2) is partially suppressed when Ulp1 is overexpressed [30]. Therefore, it seems the desumoylation (by Ulp1) or SUMO-targeted ubiquitylation (by STUbLs), specifically occurring at the NPC, could serve to modulate the activity of sumoylated proteins and/or lead to protein degradation by targeting to the proteasome. The maintenance of sumoylation levels clearly plays an important role in maintaining genome stability.

As many replication proteins are sumoylated both during normal replication and in response to replication stress, this provides another link between replication and the NPC. iPOND (isolate proteins on nascent DNA) and mass spectrometry experiments show a prevalent number of sumoylated proteins are found near the replisome in *HEK293T* cells [38]. Furthermore, studies in both yeast and human cells have shown that several replisome components themselves are sumoylated, along with replication-associated proteins. These include members of the replicative helicase (MCM2 and MCM4–6); the DNA polymerase processivity factor PCNA; several components of the RFC clamp loader complex; subunits of several polymerases (epsilon, alpha, and delta); the flap endonuclease Rad27; topoisomerases; and the ssDNA binding protein RPA [36,39,40]. The level of sumoylation of these proteins increases when treated with the DNA-damaging agent MMS [36]. Additionally, proteins that could be involved in the restart or repair of collapsed forks after encountering replication stress are also sumoylated. Some examples are Rad52, Rad59, Sgs1/BLM, Srs2, Smc5/6, Mre11, and Exo1 [36,41,42,43,44,45,46,47]. Indeed, Ubc9 and Mms21-dependent sumoylation has been shown to be important for preventing the accumulation of Rad51-dependent X structures at damaged forks [42]. The sumoylation of replication and repair proteins can lead to changes in the localization of the protein, interactions with other proteins, and activity level. Not only is sumoylation important for replication activity but, as will be outlined later in this review, several studies have shown a link between the sumoylation of specific proteins found at replication forks, the relocation of collapsed replication forks to the NPC, and maintenance of genome stability under conditions of replication stress.

## 4. Types of Barriers that Inhibit Replication and Relocate to the NPC

**Figure 1:**
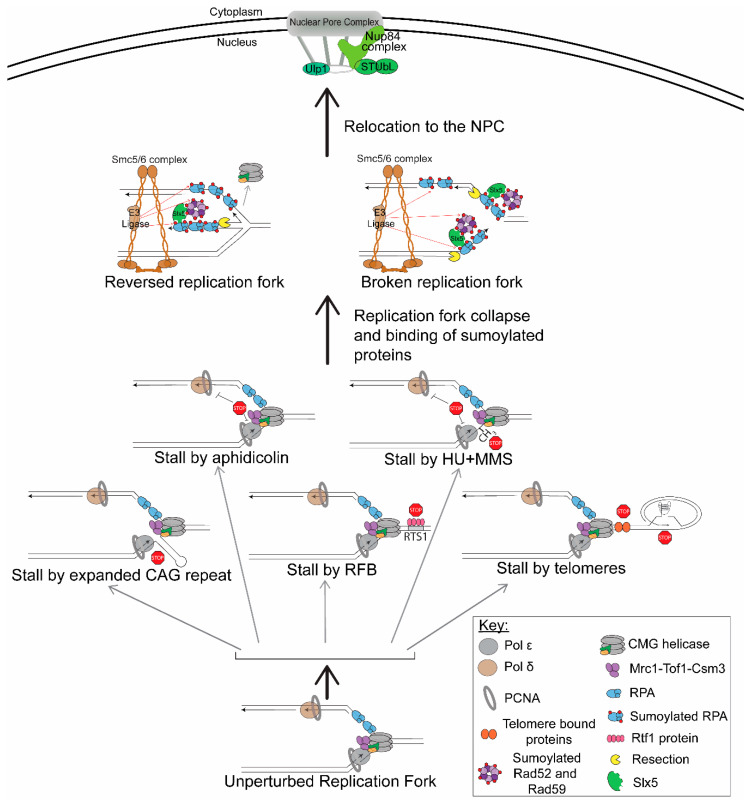
Types of replication stress that cause fork collapse and lead to relocation to the nuclear pore complex (NPC). Upon replication stress, which can be caused by many different mechanisms, normal unperturbed replication forks can stall. Several mechanisms are shown in this model: structure-forming sequences (expanded CAG repeats), protein-mediated replication fork barriers (RFBs), telomere sequences, aphidicolin, and HU + MMS. After a replication fork stalls, it can turn into a fork collapse. This is marked by dissociation of the replisome and could possibly include breaks at the replication fork; the position of the various replisome components during fork collapse at these different barriers is not known and shown for diagrammatic purposes only. For simplicity, two structures that could result from a fork collapse are shown: a reversed replication fork and a broken replication fork, although we note there are other possible structures. For example, a reversed replication fork could be cleaved by endonucleases and, in turn, result in a broken replication fork with a different structure than the one shown here. Nonetheless, both reversed and broken replication forks can be substrates for resection, which is important to generate ssDNA that binds RPA. Repair proteins, sumoylated by E3 ligases (SUMO is indicated by the red circles), bind to the ssDNA and interact with the SIM domains in Slx5 (or its homologs). This stimulates relocation to the NPC through the interaction of Slx5 with the NPC protein Nup84 (or its homologs).

Several replication fork barriers have been shown to cause relocation of the replication fork to the NPC. However, the type of replication fork barrier is important, because not all fork stalls trigger relocation to the NPC. Only in cases where the stall is severe or prolonged does relocation to the NPC occur. For example, a common way to cause fork stalling is treatment with HU, which causes the depletion of nucleotide pools. This is a transient stall in which the replisome is still intact on the replication fork; thus, the fork is not collapsed, and restart can occur after removal of the drug as long as the treatment is not prolonged [14]. Replication forks transiently stalled by HU do not relocate to the NPC [33,48]. However, prolonged treatment with HU or treatment with HU + MMS does provoke relocation [33], suggesting that fork collapse is required. Recently, several other situations in which normal replication is severely inhibited have also been shown to promote relocation to the NPC. These systems include structure-forming sequences (expanded CAG repeats) [48]; protein-mediated stalls (replication fork barriers (RFBs)) (K. Kramarz, K.S. Schirmeisen, and S. Lambert, personal communication); stalls within the telomere sequence [49]; and the inhibition of mammalian polymerases with aphidicolin [50] (Figure 1). Each of these systems will be briefly described prior to outlining the mechanistic requirements for the relocation events themselves.

NPC anchoring of collapsed forks was first shown by treatment with drugs in *S. cerevisiae*. One hour of treatment with HU does not cause relocation to the NPC, but if the treatment is extended to 2 h, relocation to the NPC does occur. In this system, a LacO array was integrated on chromosome VI near the early firing origin ARS607. The ARS607 location within the nucleus was monitored by the binding of LacI-GFP to the LacO array and was scored in relation to the nuclear periphery, which was visualized by Nup49-GFP, a nucleoporin of the NPC. When cells were released from G1 into media containing 0.2 M HU and 0.033% MMS, which leads to fork collapse near the origins, relocation to the NPC occurred (Figure 1). This was measured by colocalization of the ARS607 LacO/I-GFP foci with Nup49 in a *nup133Δ* mutant where pores cluster to one side of the nucleus [33].

Su et al. (2015) 48 showed that structure-forming sequences, which create a barrier to replication and can lead to fork collapse, cause relocation to the NPC in the budding yeast *S. cerevisiae* (Figure 1). Similar visualization methods were used as described for HU + MMS fork collapse. However, in this system, CAG trinucleotide repeats were integrated into chromosome VI with the LacO array 6.4 kb away from the CAG repeat. It is important to note that the repeat and the LacO array were separated by the early firing origin ARS607, such that they were not replicated by the same replication fork. Several repeat lengths were tested for relocation: CAG-15 does not relocate to the NPC; however, CAG-70 or CAG-130 do. The extent of relocation was dependent on both replication and repeat length, with the longer CAG-130 repeat, which causes a more severe replication barrier and more frequent fork collapse (measured by breakage rates), relocating more often. The relocation was also dependent on Nup84 and was specific to the NPC and not the nuclear envelope. This was confirmed by detecting the association of the CAG tract with Nup84 but not the inner nuclear membrane protein Mps3 using ChIP. The CAG-130 repeat interacts with Nup84 at 60 min post the alpha factor release; thus, relocation to the NPC occurs in the late-S-phase, significantly after the replication fork is predicted to first arrive at the repeat tract. Importantly, in this system, release from the pore was observed in the G2-phase, indicating that the relocation is a transient event if fork recovery is possible. Additionally, blocking the relocation pathway resulted in increased breaks at the CAG repeat, showing that it serves a protective function [48,51].

Another system in yeast, but in the fission yeast *S. pombe*, utilized a site-specific protein-mediated fork collapse and found that this type of replication fork barrier also leads to relocation to the NPC (Figure 1) (K. Kramarz, K.S. Schirmeisen, and S. Lambert, personal communication). In this system, an array of replication termination sequences (RTS1) were integrated on chromosome III. The Rtf1 protein binds to this sequence and leads to a strong replication fork barrier at a specific location (termed RFB). The Rtf1 protein is under a thiamine-repressible promoter such that the binding can be induced at specific times and monitored [52]. Similar visualization methods were used as with the CAG repeats and were first established for this system by Ait Saada et al., 2017 [53]. When the Rtf1 protein is expressed and binds to the RTS1 sequence, the RFB relocates to the NPC in the S-phase (K. Kramarz, K.S. Schirmeisen, and S. Lambert, personal communication). This is consistent with what was seen for expanded CAG repeats.

Aguilera et al. (2020) 49 utilized a telomere system in *S. cerevisiae* in which cells were deprived of telomerase to better detect fork stalling within the telomere. Without telomerase, telomeres get progressively shorter until they are critically short, which is referred to as an eroded telomere, resulting in replicative senescence. In a previous study, it was shown that eroded telomeres, which resemble single-ended breaks, relocate to the NPC [54]. They also noticed that relocation to the NPC occurred very early after telomerase inactivation and, thus, prior to erosion. They inferred that this could correspond to telomeric replication stress and fork stalling [49]. Fork stalling can occur quite frequently within the telomere sequence due to its ability to form DNA secondary structures (G-quadruplexes) and the many telomere-bound proteins [55,56,57,58] (Figure 1). Aguilera et al. utilized this observation in order to monitor the nuclear location of fork stalls occurring within telomeres. To visualize the stalled forks within the telomere they used, Cdc13-YFP foci (indicating the telomere sequence) colocalized with Rfa1-CFP foci (indicating stalled forks) [59] in cells that were telomerase-deficient (*est2Δ*). The position of the stalled fork was scored in relation to the nuclear periphery, which was visualized by the nucleoporin Nic96-RFP. The stalled replication fork relocalized to the nuclear periphery in the late-S-phase [50,54], consistent with the finding for expanded CAG tracts [48]. Disruption of a nuclear pore basket protein, Nup1, either through fusion of the bacterial DNA-binding protein LexA (*nup1-LexA*) or using a mutant with a C-terminal truncation (*nup1ΔCt*), led to a decrease in relocation of telomeric stalled forks to the nuclear periphery [49], showing that the NPC specifically is important for the relocation. The Nup1 dependency was recapitulated in cells with the CAG-130 repeat, with wild-type telomerase, further supporting that functional NPC components are required for the relocation of forks stalled at multiple types of difficult-to-replicate sequences [49].

It is important to highlight that the relocation of collapsed forks is not a phenomenon unique to budding and fission yeast but is conserved in mouse and human cells. Stalled replication forks in mouse embryonic fibroblasts that were treated with aphidicolin for 2 or 4 h associated with multiple nuclear pore components (personal communication, Dr. Anja Bielinsky) (Figure 1). For human cells, IMR90 primary fibroblasts and U-2OS osteosarcoma cells also show the relocation of stalled or collapsed replication forks to the nuclear periphery [50]. In this system, the cells were treated with aphidicolin, and the replication fork was visualized using an RFP-tagged chromobody against PCNA (RFP-PCNA-CB). Specifically, late replication foci were visualized using super-resolution images of RFP-PCNA-CB and FANCD2 colocalization (using immunofluorescence staining of fixed cells), and these foci were shown to relocate to the nuclear periphery [50,60]. The presence of FANCD2, a member of the Fanconi Anemia (FA) pathway, late in replication shows that this is probably a collapsed fork, rather than just a stalled replication fork. The FA pathway has been shown to be important for the restart of collapsed replication forks and is not needed for the restart of stalled replication forks [60]. Although FANCD2 is found enriched at both stalled and collapsed replication forks [61], the fact that the foci are late replicating points to more severe replication stress. Across species and types of replication fork barriers, it is now clear that the NPC is an area that collapsed replication forks move to during the S-phase, and therefore, the mechanisms and purpose of the relocation should be investigated.

## 5. Sumoylation Requirements for Collapsed Fork Relocation to the NPC

Although the nuclear periphery has emerged as an important site of repair for collapsed replication forks, it is not specific to collapsed forks. Relocation to the nuclear periphery was first observed for persistent DSBs and eroded telomeres in *S. cerevisiae* [33,62,63,64] and has since been observed for induced heterochromatic DSBs in *Drosophila* [65,66] and induced DSBs in the rDNA in *S. cerevisiae* [67]. Additionally, DSBs induced in the pericentric heterochromatic DNA in mammalian cells were shown to move to the periphery of the heterochromatic domain [68]. It is important to note that, in the case of induced DSBs used for the studies cited above, the break is difficult to repair due to either repeated cutting, lack of a nearby repair template, or a heterochromatic state. More easily repaired DSBs do not relocate to the nuclear periphery in yeast [33,63]

Mechanistically, there are several differences between collapsed fork and DSB relocation to the nuclear periphery. One key difference is the timing of relocation and the specific site they relocate to. In *S. cerevisiae*, a persistent DSB can relocate to both the NPC and the nuclear envelope in both the G1 and S-phases [69]. Heterochromatic DSBs in *Drosophila* also relocate to both the NPC and the nuclear envelope [65], and breaks in the rDNA in *S. cerevisiae* relocate to the NPC in both the G1 and S-phases [67]. In contrast, structure-forming CAG repeats in *S. cerevisiae* [48] only relocate during the S-phase in a replication-dependent manner and interact with the NPC but not the nuclear envelope [48]. These differences in timing and, especially, anchorage sites suggest potentially distinct pathways to relocate collapsed forks and target them specifically to the NPC.

Despite these distinct differences, there are several similarities in the mechanisms employed for relocation for DSBs and collapsed forks. One key similarity is that, consistent with previous DSB [69,70] and eroded telomere models [54,71], new data from a recent study in *S. cerevisiae* shows that the mechanism of relocation for collapsed forks is dependent on sumoylation (Figure 1). Mutations of SUMO itself, *smt3*–*331*, led to less relocation to the NPC of collapsed forks caused by structure-forming CAG repeats [51]. It was shown that only monosumoylation is required, as a mutant defective for the formation of polysumo chains (*smt3*–*3KR*) [72,73] did not impair relocation [51]. This is a key distinction from persistent DSBs where monosumoylation was not sufficient for relocation to the NPC [69]. Consistent with the requirement for sumoylation, the SUMO ligase responsible for adding SUMO to target proteins was also shown to be required for relocation: Mms21 (part of the Smc5/6 complex), but not Siz1 and Siz2 for the structure-forming CAG repeat [51]. The latter is another distinction from DSBs, as both Mms21 and Siz2 were required for the relocation of persistent DSBs to the NPC, which is consistent with the requirement for polysumoylation [51,69]

## 6. How Does Sumoylation Mediate the Movement of Collapsed Forks to the NPC?

The yeast Slx5/8 STUbL complex localizes to the NPC via interaction with Nup84 [33]. Slx5 has SUMO-interacting motifs (SIMs) that allow it to interact with SUMO and, thus, whatever proteins are sumoylated [73,74,75]. Deletion of the four main SIM domains in Slx5 impairs the relocation of structure-forming CAG repeats [51], providing evidence that Slx5’s interaction with SUMO is required for the relocation. This suggests that movement is mediated by sumoylated proteins bound at the collapsed fork interacting with the SIM domains in Slx5, which in turn interacts with Nup84 at the NPC. A tagged Slx5-mCherry protein was shown to colocalize with the CAG repeat both before and after relocation to the NPC [51], providing physical evidence that Slx5 is interacting with sumoylated proteins on the collapsed fork to facilitate the movement (Figure 1). STUbLs have also been shown to anchor persistent DSBs, heterochromatic DSBs, and eroded telomeres to the NPC [48,65,69]. However, one subtle difference is that Slx5 was critically important for collapsed fork relocation to the NPC in the S-phase and only partially required for persistent DSB relocation in the S-phase but required for G1 [69].

Since sumoylation is required for the relocation of collapsed forks to the NPC, an important question is what are the specific sumoylation targets? This has been most extensively studied in our recent work with the structure-forming CAG repeat. We identified Rad52, Rad59, and RPA as sumoylated proteins required for relocation of the collapsed fork caused by a CAG repeat to the NPC (Figure 1) [51]. Mutating the sumoylation sites on Rad52 (*rad52–3KR*) and Rad59 (*rad59–2KR*) [76] or RPA (*rpa-6KR* or *rfa1–4KR*) [77] led to a decrease in relocation. This decrease was more severe in a mutant that abolished the sumoylation of all three of these protein complexes (Rad52, Rad59, and all three subunits of RPA) [51]. Therefore, the combined sumoylation of all three repair proteins is required for optimal relocation. Interestingly, even though Rad59 appears to have a minimal role in recombination compared to Rad52 [76], the sumoylation of both proteins was required for optimal relocation of the collapsed fork to the NPC, revealing an important and novel role for Rad59. The sumoylation of Smc5, which is a part of the Smc5/6 complex that binds collapsed forks [78], also contributed [51]. To further support the sumoylation hypothesis, SUMO was fused to each of the proteins shown to play a role, and relocation to the NPC was assessed over a time course (*rad52-smt3 rad59-smt3*, *rfa1-smt3,* and *smc5-smt3*) [76]. In all cases, relocation happened earlier in the S-phase than in wild-type cells, showing that the sumoylation of these proteins is promoting the relocation [51]. Notably, Rad52, Rfa1, and Smc5 were all shown to interact with the CAG repeat prior to relocation by either fluorescence microscopy or ChIP [48,51]. This not only provides physical evidence to support that these proteins are at the collapsed fork but, also, since Smc5 is present prior to relocation, this would bring the SUMO E3 ligase Mms21 into the required proximity to sumoylate proteins at the collapsed fork and, subsequently, lead to relocation to the NPC.

For significant amounts of sumoylated RPA to bind at the collapsed fork and facilitate relocation to the NPC, there needs to be processing at the fork. Indeed, resection is required in budding yeast, as well as for the relocation of heterochromatic DSBs in *Drosophila* (Figure 1) [51,79]. Additionally, in mammalian cells, DSBs induced in pericentric heterochromatic DNA also require resection in order to relocate to the periphery of the heterochromatic domain [68]. Initial short resection by the Mre11 exonuclease activity is required for relocation in the yeast system, which is supported by the fact that Mre11-mCherry colocalizes with the CAG repeat prior to relocation [51]. In *S. cerevisiae,* long-range resection is also required, as the deletion of both Exo1 and Sgs1 (but neither alone) impaired relocation [51]. It is clear that processing of some kind is required at the collapsed fork. The generation of ssDNA provides a binding platform for sumoylated proteins, including RPA, that facilitate the relocation to the NPC.

## 7. Additional Requirements for Collapsed Fork Relocation to the NPC

In *S. cerevisiae,* the deletion of Rad51 did not impair relocation, and Rad51 did not colocalize with the CAG-130 repeat until after relocation to the NPC occurred [51]. This exclusion of Rad51 from the CAG-collapsed fork is consistent with observations for heterochromatic DSBs in *Drosophila*, in which Rad51 does not get recruited to breaks until anchoring at the nuclear periphery has occurred [65]. Similarly, in mammalian cells, Rad51 is not recruited to DSBs induced in the pericentric heterochromatic DNA until the break moves to the periphery of the heterochromatic domain [68] and DSBs in rDNA also relocate to outside of the nucleolus prior to interacting with Rad51 [80]. Therefore, the exclusion of Rad51 at the early stages of damage seems to be a commonly used mechanism. Sumoylation was shown to prevent the recruitment of Rad51 for heterochromatic DSBs, rDNA DSBs, and collapsed forks caused by CAG repeats [51,65,80]. Using non-sumoylatable mutants, we recently identified the specific SUMO target that prevents Rad51 recruitment as RPA [51]. Interestingly, though Rad52 and Rad59 sumoylation contributed to the relocation, only RPA sumoylation was responsible for excluding Rad51 binding to the collapsed fork before anchoring at the NPC occurs. This result implies that RPA sumoylation may alter its interaction with Rad52 (which loads Rad51) or the kinetics of the RPA-Rad51 exchange to disfavor formation of the Rad51 filament. It will be interesting to investigate this process at a mechanistic level in the future.

Sumoylation requirements have not been directly tested in the mouse and human cell collapsed fork systems, but in both systems, there is a checkpoint requirement for relocation. In mouse embryonic fibroblasts treated with aphidicolin, ATR inhibition led to a delay in the interaction between the collapsed replication fork and NPC components, implying a delay in relocation (personal communication, Dr. Anja Bielinsky). In human U2OS and IMR90 cells treated with aphidicolin, replication forks (visualized by RFP-PCNA-CB foci, as outlined) were shown to localize to the nuclear periphery, but when ATR was inhibited, they no longer did [50]. Additionally, the relocation of persistent DSBs to the NPC in *S. cerevisiae* and heterochromatic DSBs in *Drosophila* required both Mec1/ATR and Tel1/ATM, respectively [33,71,79]. Deletion of Mec1 or Tel1 individually did not impair relocation of the CAG-130 tract to the NPC [48]; however, recent experiments have shown that the checkpoint is required, as the deletion of other checkpoint proteins impairs relocation, though the details remain to be elucidated (M. Johnson, J.M. Whalen, A. Kim, and C.H. Freudenreich, unpublished data). Across species, it appears that the checkpoint is indeed required for the relocation of collapsed replication forks to the nuclear periphery.

Recent experiments in human cells have elucidated a potential physical mechanism for the movement of collapsed forks in the nucleus. The relocation of aphidicolin-induced collapsed forks to the periphery in U2OS cells is dependent on the polymerization of nuclear F-actin. Replication stress, induced by HU or aphidicolin, led to an increase in F-actin polymerization, visualized by phalloidin [50]. When cells were treated with Latrunculin B (LatB), an inhibitor of actin polymerization [81], the RFP-PCNA-CB foci marking the collapsed forks no longer moved to the nuclear periphery. Late-replication foci, which colocalize with FANCD2 [82], associated with actin fibers and moved along the actin fibers to the nuclear periphery [50]. This finding is consistent with previous studies of heterochromatic DSBs in *Drosophila* that require nuclear F-actin and myosin for directed movement to the nuclear periphery [83]. These data provide a mechanism for how the movement of DSBs and collapsed forks within the nucleus is occurring. It is unknown whether similar requirements are necessary in the much smaller yeast nucleus; however, the kinesin-14 motor protein complex was shown to be required for movement of subtelomeric and nontelomeric DSBs to the NPC in yeast [84,85]. The damaged DNA was observed to move along DNA damage-inducible intranuclear microtubule filaments (DIMs) to the nuclear periphery, and this movement promoted break-induced replication (BIR) [85]. This process has not been investigated for collapsed forks; however, the similarities in relocation to the NP, as well as between BIR and fork restart (both Rad51/Rad52-dependent strand invasion events), could point to similar mechanisms being involved.

## 8. Why Do Collapsed Replication Forks Go to the NPC?

The SUMO-mediated relocation of collapsed forks to the NPC appears to be an important protective mechanism to maintain genome stability. Several systems outlined in this review revealed increased genome instability when the collapsed fork does not relocate to the NPC. For CAG repeats, inhibiting relocation by deleting or mutating key genes that are required for relocation led to increased chromosome breakage (*nup84Δ*, *slx5Δ*, *smt3–331*, and *mms21–11*) and increased repeat instability (*nup84Δ* and *slx5Δ*) [48,51]. For collapsed forks caused by aphidicolin in human cells, inhibiting relocation to the nuclear periphery by inhibiting F-actin polymerization led to decreased replication fork speed, suppressed fork restart assessed using chromatin fiber assays, an extended S-phase, increases in micronuclei, and increases in anaphase abnormalities [50]. This points to the nuclear periphery being an area that maintains genome stability and promotes fork restart, which is consistent with the studies in yeast.

**Figure 2:**
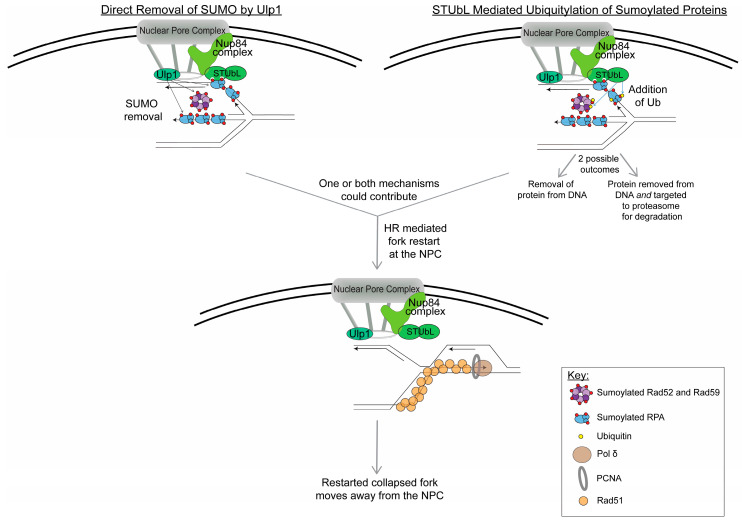
Model for events happening at the NPC to restart collapsed replication forks. Based on current data, the regulation of sumoylated proteins on the replication fork located at the NPC could occur by two mechanisms: (1) direct removal of SUMO by Ulp1 (which is bound to the NPC) and (2) STUbL-mediated ubiquitylation of sumoylated proteins (ubiquitin is indicated by yellow circles). It is important to note that both mechanisms could be happening at the same time at the collapsed replication fork. The STUbL-mediated ubiquitylation could either result in removal of the sumoylated proteins from the replication fork or it could target the sumoylated proteins to the proteasome for degradation or both. The removal and/or degradation of sumoylated proteins from the stalled replication fork is presumed to facilitate fork restart. Current data points to Rad51-dependent HR-mediated fork restart occurring while the collapsed replication fork is at the NPC; however, other outcomes at the NPC are possible, such as the resolution of HR intermediates or a converged fork. In this model, after the collapsed fork is restarted, it no longer remains at the NPC.

Data also show that a major purpose for relocation to the NPC is to restrain recombination until at the NPC. In *S. cerevisiae,* Rad51 is excluded from the collapsed fork caused by expanded CAG repeats until the collapsed fork is at the NPC [51]. The purpose of this exclusion could be to restrain recombination at the early stages of fork stalling and then promote Rad51-mediated HR-dependent fork restart at persistently stalled collapsed forks that have relocated to the NPC (Figure 2). Additionally, the CAG repeat instability observed when relocation is impaired, occurs via a Rad52-dependent mechanism [48]. Thus, relocation helps to control aberrant Rad52-dependent recombination at the stalled replication fork, perhaps in favor of the more accurate Rad51-mediated fork restart pathway. In-line with this data, tethering stalled forks within telomere sequences to the NPC in *S. cerevisiae* prevented sister chromatid recombination (SCR) and favored fork restart [49]. Interestingly, in mammalian cells, DSBs induced in the heterochromatin that fail to relocate to the periphery of the heterochromatic domain recruit Rad52 [68]. Again, these data point to a mechanism to inhibit aberrant recombination at the early stages of damage detection, with anchoring at the NPC providing a mechanism to release that inhibition if needed.

## 9. Gaps in Knowledge

There are several unanswered questions regarding the relocation of collapsed forks to the NPC. One question is why a collapsed fork causes relocation but a stalled fork does not, and what exact structure is relocating? We know that resection needs to occur for relocation to happen [51]. This means the fork needs to be in a state in which it is a substrate for resection. Two examples that could be substrates for resection are a broken fork and a reversed fork (Figure 1). A broken fork is similar, yet distinct, from a DSB and, thus, could elucidate why there are overlapping yet distinct mechanisms of relocation to the nuclear periphery for collapsed forks and DSBs. More work needs to be done to determine what exact structure, that is distinct from a stalled fork, is needed for the relocation of a collapsed fork to the NPC.

Another question is what role nuclear pore proteins are playing in the relocation process. We know that mutation of the Nup84 Y-complex leads to less relocation in several systems. Since Slx5, which is required for the relocation of CAG repeats and several DSB models, interacts with Nup84 [33], the Nup84 Y-complex could act as an anchor to tether the fork to the NPC. However, the mutation of nuclear basket proteins, such as Nup60 for collapsed forks caused by CAG repeats (X.A. Su and C.H. Freudenreich, unpublished data) or Nup1 for collapsed forks within telomeres or CAG tracts [49], also impairs relocation. What role is the nuclear basket playing in relocation? Aguilera et al. noted that the C-terminal domain of Nup1 interacts with the karyopherin Kap60-Kap95-cargo complex [86,87]. This interaction is proposed to help guide cargo (e.g., proteins being transported through the nuclear pore) to the correct site within the nucleus [87]. For example, the deletion of the human homolog of Nup1, Nup153, leads to a defect in the nuclear import of 53BP1 [88]. This leads to a delay in DNA repair, impairs cell survival after IR treatment, and increases in DNA damage caused by replication stress [88]. Moudry et al. showed that the C-terminal domain of Nup153 is required for 53BP1 nuclear import. Additionally, the deletion of Nup153 impairs the sumoylation of 53BP1, which is needed for its accumulation at DSBs [89]. It is possible that the interaction between Nup1 and the Kap60-Kap95 cargo complex could facilitate the loading of cargo on the collapsed replication fork. If this process is critical for anchoring, a defect could result in impaired relocation to the NPC. Future work will help elucidate the roles for different components of the nuclear pore complex in the relocation and anchoring of collapsed forks.

Lastly, what occurs at the NPC to promote genome stability? One hypothesis is that the removal of sumoylated proteins, or SUMO itself, at the NPC is needed in order for restart to occur, as there is evidence that sumoylation helps restrain the recombination at stalled forks [90]. The NPC is a center for sumoylation regulation, and there are two key proteins in budding yeast that could facilitate this removal: the Ulp1 SUMO protease and the Slx5/8 STUbL complex (Figure 2). Ulp1 would result in the direct removal of SUMO from proteins, whereas Slx5/8 would ubiquitylate the sumoylated proteins, resulting in removal of the protein from the chromatin and potential targeting of the sumoylated protein to the proteasome for degradation. A potential example of the latter pathway was shown in Su et al., in which sumoylated Rad52 degradation that occurred under fork collapse conditions (HU + MMS) was dependent on Slx8. This part of the pathway has yet to be completely elucidated, but it appears that the removal of SUMO and/or sumoylated proteins at the NPC could allow for Rad51-mediated HR fork restart. Despite this evidence, the question remains if all collapsed replication forks need to relocate in order for fork restart to occur. Or is it only in certain conditions that we do not fully understand yet? Events that are occurring at the NPC could also promote the resolution of HR intermediates that arise at the collapsed fork or from convergence with another replication fork. These questions will be very interesting to follow up on in the future. 

Collapsed forks caused by structure-forming repeats, protein-mediated stalls, DNA damage, or altered replication programs are naturally occurring events prevalent in the genome that must be repaired in a timely and efficient manner. There are diverse mechanisms that could be used to repair collapsed forks caused by these various replication impediments. There remains a gap in the knowledge regarding how these mechanisms function and the choices between them. Therefore, it is important to continue further investigating this pathway as relocation to the NPC is utilized across species and types of DNA damage to facilitate repair and maintain genome stability.
